# Protein-Bound Uremic Toxins: New Insight from Clinical Studies

**DOI:** 10.3390/toxins3070911

**Published:** 2011-07-20

**Authors:** Sophie Liabeuf, Tilman B. Drüeke, Ziad A. Massy

**Affiliations:** 1 INSERM ERI-12 (EA 4292), Amiens 80000, France; Email: liabeuf.sophie@chu-amiens.fr (S.L.); tilman.drueke@inserm.fr (T.B.D.); 2 Clinical Research Centre-Division of Clinical Pharmacology, Amiens University Hospital, Amiens 80000, France; 3 The Jules Verne University of Picardy, Amiens 80000, France; 4 Division of Nephrology, Amiens University Hospital, Amiens 80000, France

**Keywords:** uremic toxins, chronic kidney disease, clinical studies, indoxyl sulfate, *p*-cresyl sulfate

## Abstract

The uremic syndrome is attributed to the progressive retention of a large number of compounds which, under normal conditions, are excreted by healthy kidneys. The compounds are called uremic toxins when they interact negatively with biological functions. The present review focuses on a specific class of molecules, namely the family of protein-bound uremic toxins. Recent experimental studies have shown that protein-bound toxins are involved not only in the progression of chronic kidney disease (CKD), but also in the generation and aggravation of cardiovascular disease. Two protein-bound uremic retention solutes, namely indoxyl sulfate and *p*-cresyl sulfate, have been shown to play a prominent role. However, although these two molecules belong to the same class of molecules, exert toxic effects on the cardiovascular system in experimental animals, and accumulate in the serum of patients with CKD they may have different clinical impacts in terms of cardiovascular disease and other complications. The principal aim of this review is to evaluate the effect of *p*-cresyl sulfate and indoxyl sulfate retention on CKD patient outcomes, based on recent clinical studies.

## 1. Introduction

Kidney function impairment leads to the progressive retention of a large number of compounds which, under normal conditions, are excreted via the urinary tract [1,2]. Owing to this accumulation, the retained molecules are called uremic retention solutes or, as adopted by the EUTox group, *uremic toxins.* It is notable that all these compounds exhibit characteristic biological and/or biochemical activities. 

The uremic toxins can be classified according to their molecular weight (MW) and their protein-binding ability [3], the most convenient classification being (1) small MW water-soluble compounds; (2) protein-bound compounds; and (3) larger MW compounds, the so-called “middle molecules” [4].

Toxic effects seem to be induced by compounds which are difficult to remove by dialysis. This is particularly true for the second group, namely the protein-bound uremic toxins which are poorly eliminated by the commonly used dialysis techniques. The present review will focus on this specific class of molecules.

Our group and others have recently studied two prototypes of protein-bound uremic retention solutes, *p*-cresyl sulfate and indoxyl sulfate. As shown by us and others, their serum levels are elevated in advanced stages of chronic kidney disease (CKD) and correlate with glomerular filtration rate [5,6,7].

In patients with CKD, cardiovascular disease (CVD) is highly prevalent [8]. In addition to classical Framingham risk factors, uremic toxins can be considered as nontraditional risk factors in this population. Thus *p*-cresyl sulfate and indoxyl sulfate have been shown to exert toxic effects *in vitro* [9]. Moreover, results obtained in different CKD patient cohorts (both in hemodialysis and in predialysis patients) have identified these two uremic toxins as emerging mortality risk factors [5,6,7,10,11,12]. 

The aim of the present review is to provide new insight into the respective impacts of *p*-cresyl sulfate and indoxyl sulfate retention for the outcomes of patients with CKD, based on recent clinical studies.

## 2. Indoxyl Sulfate and *p*-Cresyl Sulfate Formation

The action of intestinal bacteria on undigested proteins gives rise to a variety of indoles and phenols that are conjugated by the liver before being excreted by the kidney. Indoxyl sulfate and *p*-cresyl sulfate are examples of these compounds. Their accumulation in the uremic state has a negative impact on many body functions. They are prototype members of the large group of protein-bound uremic toxins [4].

Briefly, tryptophan is metabolized into indole by intestinal bacteria which, after intestinal absorption, is further converted to indoxyl sulfate in the liver. *P*-cresol emanates from the metabolism of the amino acids tyrosine and phenylalanine by the intestinal flora. These amino acids are generated from dietary proteins and metabolized to 4-hydroxyphenylacetic acid, which is then decarboxylated to *p*-cresol [13,14]. During its passage through the intestinal mucosa, a cytosolic sulfotransferase metabolizes *p*-cresol to *p*-cresyl sulfate [15]. *P*-cresol is present in the circulation largely in the form of its sulfate conjugate, *p*-cresyl sulfate. 

In many clinical studies, authors reported data on *p*-cresol; however, *p*-cresol was determined after acidification, so that the concentrations measured were in fact those of the main retention solute, *p*-cresyl sulfate as each molecule of sulfate which is broken down by hydrolysis will generate one molecule of *p*-cresol [16]. 

The serum levels of these two toxins can be evaluated by reverse phase high performance liquid chromatography. This method evaluates 2 forms, namely the free and the total molecule [17]. 

## 3. Indoxyl Sulfate, *p*-Cresyl Sulfate and Renal Toxicity

Both toxins are excreted by the kidneys via proximal tubular secretion. Consequently, they accumulate in the blood of patients with impaired renal function [18,19]. Moreover, they cannot be efficiently removed by conventional haemodialysis, due to their high binding affinity for albumin. 

Our research group and others have demonstrated in a cohort of patients with different CKD stages that total and free indoxyl sulfate and total and free para-cresyl sulfate serum levels are elevated in the advanced stages of CKD and that they correlate with glomerular filtration rate in pre-dialysis patients [5,6,7].

In addition, some data suggested that they could have a negative impact on the kidney. Indeed, it has been demonstrated that indoxyl sulfate plays a role in the progression of CKD by inducing an inflammatory reaction, with enhanced expression of profibrotic cytokines such as transforming growth factor beta 1 (TGF 1) [20,21]. Moreover, administration of indoxyl sulfate to 5/6-nephrectomized rats accelerated the development of renal fibrosis [22], and its administration to hypertensive rats reduced the expression of klotho and promoted cell senescence accompanied by renal fibrosis [23].

## 4. Indoxyl Sulfate, *p*-Cresyl Sulfate and Vascular Toxicity

These two toxins may also have a role in the development of uremia-related cardiovascular disorders. 

Although it has been shown that *p*-cresol can affect endothelial barrier function, endothelial cell proliferation and wound repair [9,24], it remains to be seen whether *p*-cresyl sulfate also exerts harmful effects on vascular cells *in vitro*. If so, not only *p*-cresol but also its sulfate conjugate would exert direct actions on the cardiovascular system. Indeed, Schepers *et al*. observed that *p*-cresyl sulfate (but not *p*-cresol) had pro-inflammatory effects on non-stimulated leukocytes *in vitro*—suggesting that *p*-cresyl sulfate may contribute to the propensity of renal patients towards vascular damage [25].

In addition to its profibrotic effects, indoxyl sulfate may also favor the development of CVD by inhibiting endothelial cell repair and promoting the proliferation of vascular smooth muscle cells [9]. Indoxyl sulfate may play an important role in endothelial dysfunction via the generation of oxidative stress and induction of endothelial senescence [26]. A recent paper suggested that contrasting with CKD conditions (in which indoxyl sulfate has pro oxidant properties), under normal physiological conditions, it could act as an antioxidant. [27]. The latter experimental and clinical data suggest a role of these uremic toxins in vascular dysfunction in CKD patients. This hypothesis is corroborated by recent personal clinical studies. Thus, we demonstrated a weak relationship of serum-free and total *p*-cresyl sulfate with vascular calcification in 139 patients with different CKD stages, whereas no relationship existed with arterial stiffness, as evaluated by pulse wave velocity measurements. Similarly, serum-free and total indoxyl sulfate levels correlated with aortic calcification, and in addition also with vascular stiffness [5,6].

It is noteworthy that the concentration of such hydrophobic uremic toxins can be reduced by medical intervention. Thus, the oral charcoal adsorbent AST-120 (Kremezin, Kureha Chemical Industry, Tokyo, Japan) has been shown to be capable of adsorbing uremic toxins and thereby attenuating the oxidative stress generated by them [28]. In a study in which AST-120 was administered to pre-dialysis CKD patients for two years, a significant reduction in carotid intima-media thickness and PWV was reported in the AST-120 group, when compared with the non-AST 120 group [29]. Moreover, a recent study in 40 CKD patients showed that the administration of AST-120 led to an improvement in endothelial dysfunction in patients with CKD, in association with a decrease in serum indoxyl sulfate levels and a reduction in circulating markers of oxidative stress [26]. As AST 120 is not a specific adsorbent of indoxyl sulfate, presumably the serum concentrations of other organic compounds including *p*-cresyl sulfate levels have been decreased simultaneously with the decrease of serum indoxyl sulfate [26].

## 5. Indoxyl Sulfate, *p*-Cresyl Sulfate and Clinical Outcomes

Because of the available experimental and clinical evidence in favor of the involvement of the two toxins in the vascular damage observed in CKD, possible associations with cardiovascular outcomes and mortality have been sought in recent years. 

In a personal study, we have indeed been able to show that higher serum indoxyl sulfate (both free and total molecules) was associated with an increase overall and cardiovascular mortality in patients at different CKD stages [6]. This effect was independent of traditional risk factors, vascular stiffness and aortic calcification. In contrast, two other studies, one in dialysis patients and the other in predialysis patients, did not find an association between indoxyl sulfate levels and overall mortality [12,30]. However, in these two studies, the mortality rate was low and the main objective was not to examine overall mortality.

The impact of serum *p*-cresol/*p*-cresyl sulfate concentrations on outcomes has been studied in two distinct patient cohorts, namely end stage renal disease patients and predialysis CKD patients. 

*Hemodialysis patients*. In 175 prevalent chronic hemodialysis patients, free, but not total, *p*-cresol concentrations have been found to be related to mortality in unadjusted and adjusted models [11]. Moreover, in another group of non-diabetic hemodialysis patients, free *p*-cresol was significantly associated with cardiovascular disease [10]. Finally, Lin *et al.* recently showed that serum-free and total *p*-cresol levels were strongly related to cardiovascular disease in dialysis patients [12]. 

*Predialysis patients.* Meijers *et al*. have recently demonstrated an association of serum-free *p*-cresol with cardiovascular events in a cohort of 499 patients with mild-to-moderate CKD [7]. An interesting finding was that the association was independent of glomerular filtration rate and Framingham risk factors. Other authors found that high serum *p*-cresyl sulfate levels were associated with all cause mortality risk in a group of 268 predialysis patients [30]. In the same vein, we observed in a group of patients with different stages of CKD that serum-free, but not total, *p*-cresyl sulfate was associated with overall and cardiovascular mortality risk independently of other risk factors [5].

Taken together, results from CKD cohorts in different centers and countries suggest different impacts of indoxyl sulfate and *p*-cresol/*p*-cresyl sulfate and their total and free forms, respectively, on the relative risk of cardiovascular and all-cause mortality. Only one report suggested that both serum total and free indoxyl sulfate concentrations were associated with negative patient outcomes, whereas the majority of reports found a negative impact only from the free fraction of *p*-cresol/*p*-cresyl sulfate, but not the total fraction, with the exception of one study [12]. 

**Figure 1:**
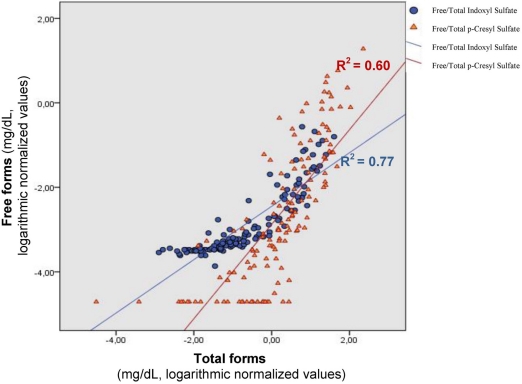
Relationships between free and total forms of indoxyl sulfate (*r*^2^ = 0.77, *p* < 0.001) and free and total forms of *p*-cresyl sulfate levels (*r*^2^ = 0.60, *p* < 0.001) in uremic serum.

To better understand these discrepancies, we compared in our cohort of 139 patients with different CKD stages, the correlation between serum levels of total and free fractions for each of these two toxins, as well as the percentage of binding defined as: (total − free)/total × 100. We found that the total and free serum concentrations of indoxyl sulfate were closely associated (*r*^2^ = 0.77, *p* < 0.001). An association also existed between the free and total serum levels of *p*-cresyl sulfate (*r*^2^ = 0.60, *p* < 0.001). However, the two associations, although being linear, differed from each other according to Wald’s test, as shown in Figure 1, indicating a different type of correlation between the free and the bound form among these two toxins.

Since we found that a higher free level of each of these 2 toxins was associated with a higher relative risk of mortality, we compared their per cent protein binding according to the median of their free fraction levels, as described in Tables 1 and 2. The results of this analysis showed that, with increasing free toxin levels, the per cent binding of indoxyl sulfate increased as well (Table 1). In contrast, the per cent binding of *p*-cresyl sulfate decreased with increasing free moiety concentrations (Table 2). These data suggest that the percentage of free *p*-cresyl sulfate levels increased differently at high toxin concentrations than the percentage of free indoxyl sulfate. This difference could play a role in the observed differences of toxicity between the two molecules.

**Table 1:** Per cent protein binding according to levels of free indoxyl sulfate divided by median level.

	**Free Indoxyl Sulfate**			*p*
	Total	≤0.038 mg/100 mL (*n* = 70)	>0.038 mg/100 mL (*n* = 69)	
Binding (%)	89 ± 8 (88.9)	83 ± 9 (86)	91 ± 5 (92)	<0.001

Data are presented as mean ± standard deviation (median). Binding percentage is defined as (Total − Free)/Total × 100.

**Table 2:** Per cent protein binding according to levels of free *p*-cresyl sulfate divided by the median level.

	**Free *p*-Cresyl Sulfate**			*p*
	Total	≤0.051 mg/100 mL ( *n* = 70)	>0.051 mg/100 mL ( *n* = 69)	
Binding (%)	91 ± 11 (95.8)	97 ± 4 (97.5)	85 ± 13 (90.2)	<0.01

Data are presented as mean ± standard deviation (median). Binding percentage is defined as (Total − Free)/Total × 100.

It is well known that indoxyl sulfate and *p*-cresyl sulfate are competitive binding substrates for the same albumin binding sites, reaching 95% of binding for both molecules [10]. However, these latter data have been observed with separation techniques [31]. Recently, the binding of *p*-cresyl sulfate and *p*-cresol with human serum albumin was studied using microcalorimetry [32]. The authors found a moderate affinity of both *p*-cresyl sulfate and *p*-cresol toward human serum albumin at 25 °C which became relatively weak at the physiological temperature of 37 °C. The binding appears to involve principally van der Waals type interactions, the binding sites of the two molecules being the same or very close. The low fraction of protein-bound toxin, 13−20%, is therefore probably insufficient to incriminate strong binding as the main cause of poor removal of these toxins by hemodialysis. Thus *p*-cresyl sulfate binding to albumin appears to be less strong than previously reported [32].

Since there is an association between the circulating levels of these uremic toxins and the relative risk of cardiovascular events and mortality in patients with CKD, it will be interesting to test the hypothesis that an active reduction of their levels should lead to a decrease in cardiovascular and global outcomes. Interestingly, it has been preliminarily reported that AST-120 given to CKD patients prior to dialysis initiation improved their overall survival rates, in comparison with CKD patients to whom AST-120 was not administered [33]. 

As pointed out above, it has long been known that AST 120 can absorb many organic compounds. According to a recent study, it is able to reduce the plasma levels of several solutes retained in the uremic state, including hippurate, phenylsulfate, indoxyl sulfate and *p*-cresyl sulfate [34]. However, the authors did not report which fraction, namely free or total, was evaluated. This does not weaken the evidence for a beneficial effect of AST-120. It only limits our ability to separate the effect on the reduction of the 2 uremic toxins under evaluation (both the free forms and the total molecules) from that on the reduction of other circulating solutes.

## 6. Conclusions

There is increasing experimental and clinical evidence in favour of the hypothesis that uremic toxins, and in particular protein-bound toxins, are involved not only in the progression of CKD, but also in the promotion of cardiovascular disease. Two protein-bound uremic retention solutes, namely indoxyl sulfate and *p*-cresyl sulfate, could play a prominent role. However, although the two of them belong to the same class of molecules, exert toxic effects on the cardiovascular system and accumulate in the serum of patients with CKD, they may have different impacts in terms of cardiovascular disease. One of the reasons for differing toxicities could be differences in the proportion of free and total fractions among the two toxins, resulting from differences in protein binding affinity and/or potential competition in protein binding. In our opinion, these hypotheses deserve to be examined by further studies. 
